# Improved lipid productivity in *Nannochloropsis gaditana* in nitrogen-replete conditions by selection of pale green mutants

**DOI:** 10.1186/s13068-020-01718-8

**Published:** 2020-04-21

**Authors:** Michela Cecchin, Silvia Berteotti, Stefania Paltrinieri, Ivano Vigliante, Barbara Iadarola, Barbara Giovannone, Massimo E. Maffei, Massimo Delledonne, Matteo Ballottari

**Affiliations:** 1 Dipartimento di Biotecnologie, Università degli Studi di Verona, Strada le Grazie 15, 37134 Verona, Italy; 2 Dipartimento di Scienze della Vita e Biologia dei Sistemi, Unità di Fisiologia Vegetale, Università di Torino, Via Quarello 15/a, 10135 Turin, Italy

**Keywords:** Microalgae, Photosynthesis, Nannochloropsis, Biofuel, Palmitic acid, Stearic acid

## Abstract

**Background:**

*Nannochloropsis gaditana* is a photosynthetic unicellular microalgae considered one of the most interesting marine algae to produce biofuels and food additive due to its rapid growth rate and high lipid accumulation. Although microalgae are attractive platforms for solar energy bioconversion, the overall efficiency of photosynthesis is reduced due to the steep light gradient in photobioreactors. Moreover, accumulation of lipids in microalgae for biofuels production is usually induced in a two-phase cultivation process by nutrient starvation, with additional time and costs associated. In this work, a biotechnological approach was directed for the isolation of strains with improved light penetration in photobioreactor combined with increased lipids productivity.

**Results:**

Mutants of *Nannochloropsis gaditana* were obtained by chemical mutagenesis and screened for having both a reduced chlorophyll content per cell and increased affinity for Nile red, a fluorescent dye which binds to cellular lipid fraction. Accordingly, one mutant, called *e8*, was selected and characterized for having a 30% reduction of chlorophyll content per cell and an almost 80% increase of lipid productivity compared to WT in nutrient-replete conditions, with C16:0 and C18:0 fatty acids being more than doubled in the mutant. Whole-genome sequencing revealed mutations in 234 genes in *e8* mutant among which there is a non-conservative mutation in the *dgd1* synthase gene. This gene encodes for an enzyme involved in the biosynthesis of DGDG, one of the major lipids found in the thylakoid membrane and it is thus involved in chloroplast biogenesis. Lipid biosynthesis is strongly influenced by light availability in several microalgae species, including *Nannochloropsis gaditana*: reduced chlorophyll content per cell and more homogenous irradiance in photobioreactor is at the base for the increased lipid productivity observed in the *e8* mutant.

**Conclusions:**

The results herein obtained presents a promising strategy to produce algal biomass enriched in lipid fraction to be used for biofuel and biodiesel production in a single cultivation process, without the additional complexity of the nutrient starvation phase. Genome sequencing and identification of the mutations introduced in *e8* mutant suggest possible genes responsible for the observed phenotypes, identifying putative target for future complementation and biotechnological application.

## Background

Microalgae are photoautotrophic organisms that can be cultivated to exploit light energy to fix CO_2_ into organic biomass. Microalgae-derived biomass can then be used for several applications, among which the production of food, high-value products and/or biofuels [1–4]. Some microalgae species indeed can accumulate high amounts of lipids, the biomass constituents with the highest energy associated [5]. Fatty acids are mainly synthesized in the chloroplast and then used as building blocks for triacylglycerols (TAGs), which are deposited in densely packed lipids bodies located in the cytoplasm of the algal cell [6]. In oleaginous algae, the lipid content varies between 20 and 70% and can reach values up to 90% of algal total dry weight under certain conditions, such as nitrogen deprivation [7]. Nutritional stress is a common strategy adopted by the microalgae research community to boost TAGs accumulation which can be converted to biodiesel by a transesterification reaction [8]. Two-phase cultivation for inducing lipid biosynthesis in microalgae is however a costly process, requiring modification of the growth medium and additional time required before biomass harvest. Species belonging to the genus *Nannochloropsis* are marine unicellular microalgae [9] considered among the most promising strains for cultivation in large scale systems, as open ponds or closed photobioreactors, for biodiesel production due to their fast growth rate, lipid accumulation (up to 65–70% of total dry weight) and ability to adapt to different irradiation conditions [5, 10, 11]. In addition, 30% of fatty acids accumulated in *Nannochloropsis* are polyunsaturated fatty acids among which eicosapentaenoic acid (EPA, 20:5ω3), one of the major omega-3 fatty acid reported to have positive effect in human health [12]. This yellow green alga belongs to the class the pico-plankton *Eustigmatophyceae*, composed by species mainly living on the coasts. The cells of *Nannochloropsis* have reduced size (3–5 μm) [9], with a single chloroplast occupying most of the cell volume [13, 14]. It shows a peculiar pigments content, presenting only chlorophyll (Chl) *a* and lacking other accessory chlorophylls such as Chl *b* or *c* while violaxanthin and vaucheriaxanthin are the most represented carotenoids [15]. The *N. gaditana* genome is available and its assembly includes nuclear (~ 29 Mbp) and organellar genomes, containing ~ 10.000 gene models [16, 17]. The availability of a genome sequence and transformation methods allow genetic engineering strategies to further improve this naturally productive species [18, 19].

Although microalgae are attractive biomass, bioproducts and biofuel producers, their photosynthetic efficiency is much lower compared to their theoretical potential [20]. Light use efficiency of microalgae in photobioreactors is indeed limited by the steep light gradient due to the strong optical density of the near-molar concentration of chlorophylls in cells [21]. This non-homogeneous light penetration results in a low productivity of the system, the inner layers being almost in the dark [21]. Mutant strains with reduced pigment content per cell resulting either from a truncated antenna size or a lower overall density of photosynthetic units per cell were reported for different species, as *C. reinhardtii, C. vulgaris, C. sorokiniana* and *N. gaditana*, being characterized by an increased productivity [22–25]. In addition, up to 80% of the light absorbed by the external layers is dissipated as heat by the activation of photoprotective processes, with consequent loss of light use efficiency and biomass productivity [26]. The photoprotective mechanism involved in the energy dissipation as heat, is known as non-photochemical quenching (NPQ), a short-time response to energy absorbed in excess, triggered by lumen acidification when the photosynthetic apparatus is saturated [27, 28].

These photosynthetic limitation influences both biomass yield and lipid accumulation. Indeed, the key challenge for the oleaginous algae is to maximize lipid production maintaining high biomass yields [5, 29]. The main strategies at industrial level to trigger lipid accumulation in microalgae are to induce nutrients starvation, especially nitrogen starvation: in these conditions cells redirect carbon metabolism into nitrogen-free lipid molecules [5]. However, this approach strongly reduces cells growth rate, affecting overall biomass and lipid productivities. Some positive results were obtained by overexpression or downregulation of transcriptional factor increasing the lipid production with moderate or even positive effects for the growth [30, 31]. However, the possibility to use genetic modified organisms (GMOs) at industrial scale is still limited by the different acceptance and legislation in the different countries, hampering the application of the promising results obtained.

In this work, we report a biotechnological approach by chemical mutagenesis to isolate *N. gaditana* strains with increased lipid productivity in absence of nutrient starvation. The strategy adopted was the selection of strains with both a reduction in cell pigmentation, to allow a better light distribution in photobioreactor, and an increased Nile red staining, as a probe for lipid accumulation.

## Results

### Mutagenesis and selection of mutant strains

**Fig. 1:**
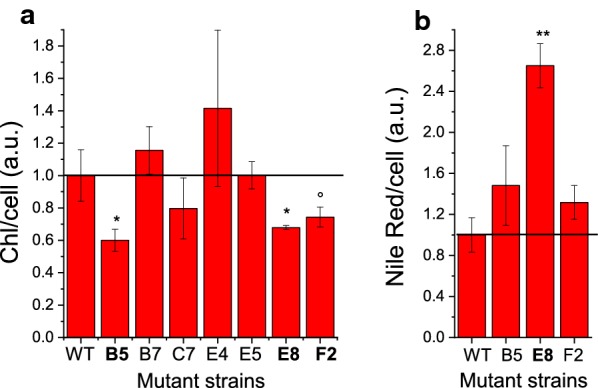
Chlorophylls per cell and Nile red staining of mutated strains. Chlorophyll content per cell (**a**) and Nile red fluorescence per cell (**b**) were normalized to the WT case. Errors are reported as standard deviation (*n* = 3), significantly different values are marked with * if *p* < 0.05 and ** if *p* < 0.01, as determined by unpaired two-sample *t* test (*n* = 3). In the case of sample marked with °*p*-value of 0.0597 was obtained

*N. gaditana* mutants were obtained by chemical mutagenesis, using the alkylating agent ethyl methane sulfonate (EMS) that inserts random single-point mutations (SNP) in the genome. Surviving colonies with a visible “pale green” phenotype in solid medium were initially selected, transfer to liquid medium and screened by measuring absorption of chlorophyll *a* (at 680 nm) and cell scattering (at 730 nm). The resulting 680/730 nm absorption ratio provides a relative indication of the chlorophyll content per cell. Seven strains were selected having a 680/730 nm absorption at least 25% decreased compared to the WT case (Additional file 1: Figure S1). These strains were then evaluated for their chlorophyll content per cell by extracting pigments, quantifying them and counting the cells: only 3 mutants showed a decreased Chl/cell ratio (Fig. 1a). These mutants were then further analyzed for their lipid content by Nile red staining: as reported in Fig. 1b, only in the case of mutant *e8*, an increased Nile red fluorescence was measured per cell. The selected mutant *e8* was thus characterized by a 30% reduction of chlorophyll content per cell and ~ 180% increase in Nile red staining, suggesting an increased lipid content.

### Photosynthetic characterization of *e8* mutant

**Fig. 2:**
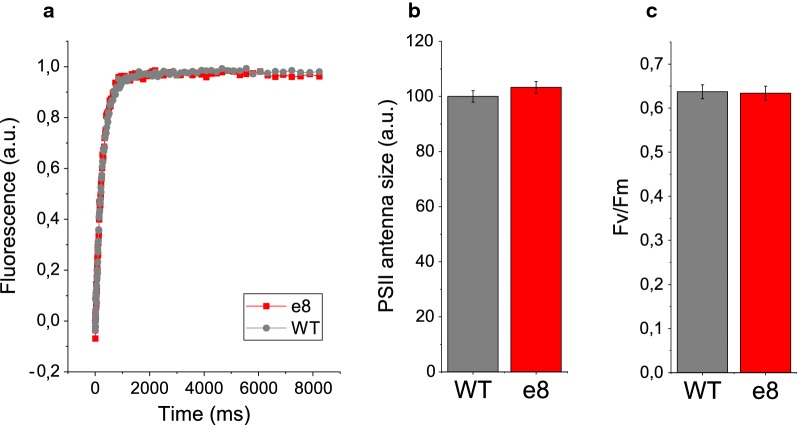
PSII functional antenna size and PSII maximum quantum yield. **a** Fluorescence induction kinetics of PSII antenna size of wild type and selected mutant. **b** PSII functional antenna size expressed as the reciprocal of the time required to reach 2/3 of the maximum fluorescence emission, τ_2/3_^−1^(%). **c** PSII maximum quantum yield calculated as (*F*_M_-*F*_0_)/*F*_M_ from basal chlorophyll fluorescence in the dark (*F*_0_) and maximum chlorophyll fluorescence induced by a saturating pulse (*F*_M_). The statistical analysis of the results obtained was performed by unpaired two-sample t-test (*n* = 4, no statistically significant difference being *p*-value = 0.09 for PSII functional antenna size and *p* = 0.78 for *F*_v_/*F*_m_ values)

**Fig. 3:**
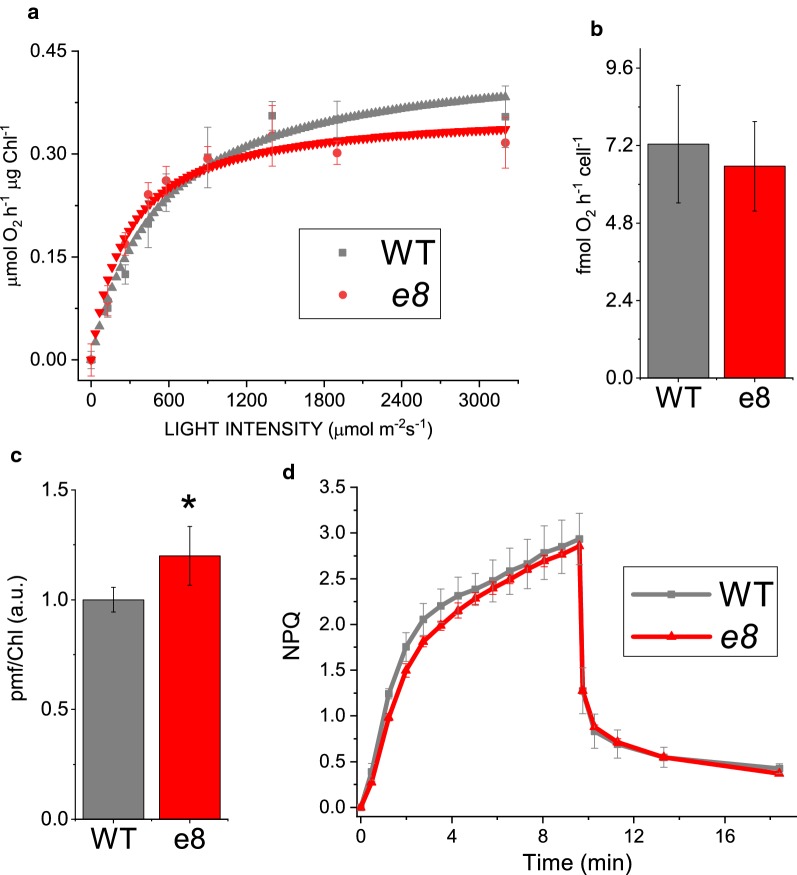
Photosynthetic parameters of e8 mutant compared to WT. **a** Net oxygen production of wild type and *e8* mutant strain normalized to chlorophyll content, measured at different actinic light intensities. Experimental data were fitted with hyperbolic function. **b** Dark respiration rate normalized to cell content. **c** Non-Photochemical Quenching (NPQ) formation and relaxation in wild type and *e8* mutated strain, actinic light 1500 μmol photons m^−2^ s^−1^. **d** Proton motive force (*pmf*) for wild type (WT) and *e8* obtained by electrochromic shift measurement (ECS) at 1000 μmol photons m^−2^ s^−1^ and normalized to the chlorophyll content. Errors are reported as standard deviation, the statistical significance of differences between WT and *e8* is indicated as * (*p* < 0.05), as determined by unpaired two-sample t-test (*n* = 4)

Pigment composition of *e8* strain was analyzed by HPLC and compared to the WT case. As reported in Table 1, the accumulation of the different carotenoid species was similar in the *e8* mutant strain compared to WT. Chl/cell reduction observed in *e8* mutant was related to the total cell content of chlorophylls but the pigments composition on a chlorophylls basis was essentially unaffected. Only in the case of zeaxanthin, a significant increase was observed in the *e8* mutant compared to WT, with a ~ 180% increase on a chlorophyll basis. In order to evaluate if the reduced Chl/cell ratio observed in *e8* mutant was related to a truncated antenna phenotype [25, 32], the functional antenna size of Photosystem II (PSII) was estimated measuring the kinetics of fluorescence induction in cells treated with the PSII inhibitor 3-(3,4-dichlorophenyl)-1,1-dimethylurea (DCMU) [33]. The inhibitor DCMU blocks the electron transport from PSII to plastoquinone pool, inducing PSII to re-emit as fluorescence the excitation energy absorbed: upon DCMU treatment, in limiting light, the capacity of light harvesting and energy transfer to reaction center of PSII is inversely proportional to the fluorescence emission kinetics (Fig. 2a) [33]. The differences in antenna size were thus quantified as the reciprocal of the time required to reach 2/3 of the maximum fluorescence (1/τ_2/3_, Fig. 2b). As reported in Fig. 2a, the *e8* mutant strain showed fluorescence induction kinetics similar to the WT case. Thus, the reduced Chl/cell ratio was not related to a reduced antenna/core complex ratio and reduced light harvesting capacity of PSII. PSII maximum quantum yield was then measured by pulse amplitude modulated fluorescence as F_v_/F_m_ (Fig. 2c). F_v_/F_m_ was not significantly different in *e8* mutant compared to WT, suggesting that the mutations introduced in *e8* were not deleterious to photosynthesis. Photosynthetic performances of *e8* were then evaluated measuring the light-dependent oxygen evolution. Net oxygen evolution rates at different light intensities are reported in Fig. 3a, normalized to the chlorophyll content and fitted with hyperbolic functions, showing no major differences between WT and *e8* mutant. Similarly, dark respiration rate of WT and *e8* was not significantly different, suggesting that mitochondrial respiration was not affected by the mutation introduced in the mutant (Fig. 3b).

**Table 1:** Pigment analysis of WT and *e8* mutant strain

	Chl/cell (%)	chl *a*	Chl/car	Vio	Vau	Anthera	Cantha	Zea	β-car
WT	100.0%	100.00	2.31	20.51	12.17	3.56	0.67	3.21	3.27
*s.d.*	13.8%	5.04	0.17	1.11	0.71	0.48	0.53	0.60	0.31
*e8*	69.6%*	100.00	2.17	19.14	13.37	3.64	1.03	5.90*	2.97
*s.d.*	2.5%	*9.16*	*0.18*	*0.94*	*0.73*	*0.50*	*0.34*	*1.32*	*0.38*

Chlorophyll content per cell (Chl/cell) was set to 100% in the case of WT. The concentration of pigments in pmol was determined by HPLC and normalized to 100 pmol of chlorophyll *a* (Chl). Violaxanthin: vio, vaucheriaxanthin: vau, β-carotene: β-Car, antheraxanthin: anthera, zeaxanthin: zea, canthaxanthin: cantha. Standard deviations (s.d.) are reported for the different values (*n* = 5 for Chl/cell values, *n* = 3 for the other values). Significantly different values are marked with * if *p* < 0.05, as determined by unpaired two-sample t-test (*n* = 3)

Light-dependent electron transport in photosynthetic organisms is coupled to proton transport across the thylakoid membrane into the lumen, which is then used by ATPase to produce ATP. Light-dependent proton motive force (*pmf)* can be estimated measuring the light-dependent electrochromic shift (ECS) of carotenoid absorption. Carotenoid absorption spectra are indeed sensitive to the membrane potential [34]. As reported in Fig. 3c, a significant increase in *pmf* was evident in *e8* mutant when exposed to actinic light, indicating an increased proton transport across thylakoid membranes. This result suggests that the reduction of Chl content per cell observed in *e8* mutant did not negatively influenced trans-thylakoid proton transport but rather increased the light-dependent *pmf*, because of possible adaptation of the photosynthetic apparatus to the *e8* mutant phenotype. Considering the increased *pmf* observed in *e8* mutant, the activation of photoprotective mechanisms triggered by lumenal ΔpH was then investigated as xanthophyll cycle activation and NPQ induction. Light-dependent zeaxanthin accumulation was measured in WT and *e8* mutant upon exposure to strong light (2500 μmol photons m^−2^ s^−1^) for 1 hour in order to induce violaxanthin de-epoxidation (Additional file 1: Figure S2): *e8* mutant strain showed a higher de-epoxidation index only in the first minutes of illumination, due to the higher accumulation of zeaxanthin at time zero compared to the WT, but on a longer time scale the zeaxanthin content was similar in the two strains. Considering the role of this xanthophyll in the photoprotective mechanisms adopted by *N. gaditana* [35, 36], we measured the NPQ induction kinetics in *e8* mutant compared to WT. As reported in Fig. 3d, the NPQ kinetics were similar in *e8* mutant compared to the WT case. NPQ induction was indeed reported to be only partially related to the xanthophyll cycle activation in *N. gaditana* [36]: the zeaxanthin content in *e8* mutant and WT, even if different in the first minutes of illumination, was likely sufficient to saturate the zeaxanthin-dependent NPQ component in both strains at the actinic light used.

In order to investigate possible different photosensitivity of the *e8* mutant compared to WT, chlorophyll bleaching kinetics were measured upon exposure to strong light (2500 μmol photons m^−2^ s^−1^). As reported in Additional file 1: Figure S3, the exposure to strong light causes in both WT and *e8* strain a similar decrease in chlorophyll absorption, with a ~ 30% chlorophyll loss after 14 h of illumination. This result demonstrates that the *e8* strain is not impaired in photoprotective mechanisms.

### Biomass and lipid productivity

**Fig. 4:**
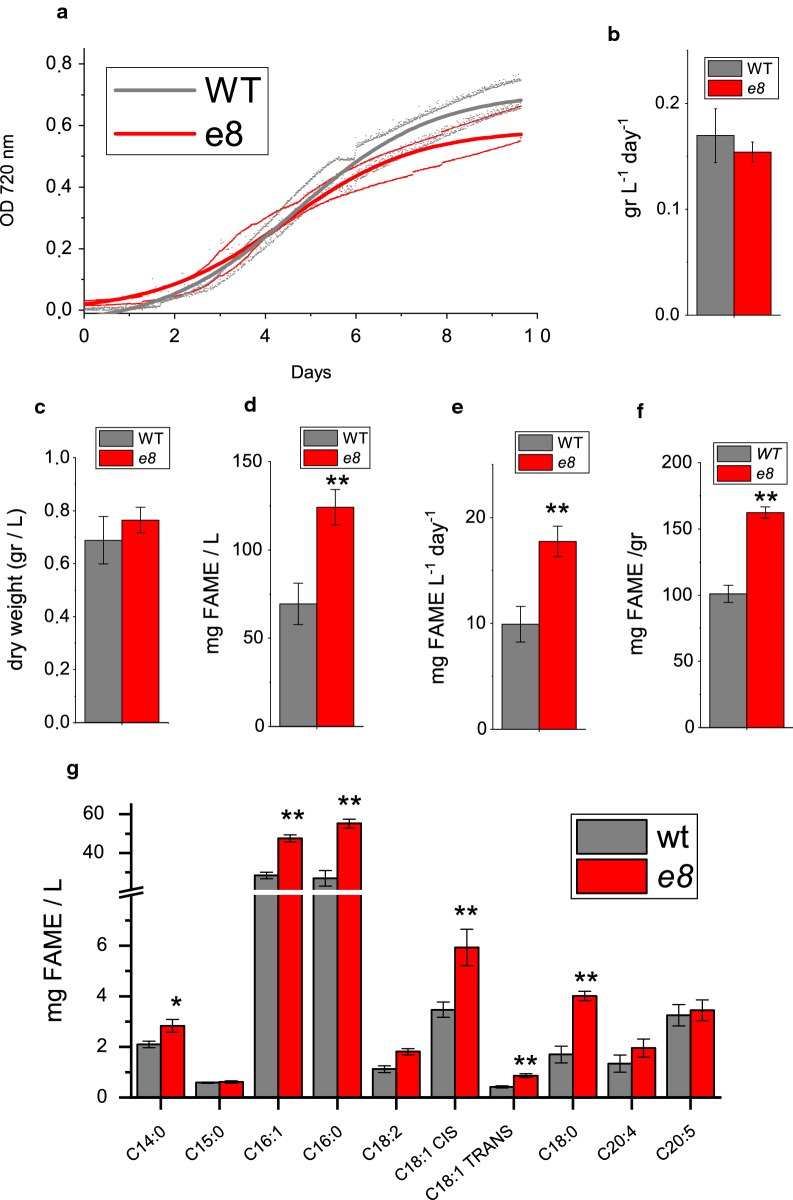
Biomass and lipid productivity of wild type and *e8* mutant. **a** Growth curves of WT and *e8* mutant obtained measuring the optical density at 720 nm. **b** Maximum daily productivity in terms of gr L^−1^ day^−1^. **c** Dry weight at the end of the growth curve (g/L). **d**, **e** FAME content in terms of mg of lipids per liters of culture (**d**) or mg of lipids per gram of dry weight (**e**). **f** FAME productivity in terms of mg of lipids per liters of culture per day. **g** Acyl chain composition of lipid fraction from WT and *e8* mutant. Errors are reported as standard deviation, significantly different values are marked with * if *p* < 0.05 and ** if *p* < 0.01, as determined by unpaired two-sample t-test (*n* = 3)

Biomass productivity, defined as biomass dry weight obtained on a daily basis, in WT and *e8* mutant strain was analyzed in 80 ml batch airlift photobioreactors illuminated with continuous white light at different irradiances, from 60 to 1500 μmol photons m^−2^ s^−1^. As reported in Fig. 4 and Additional file 1: Figure S4, increased biomass productivity was measured at 60 and 200 μmol photons m^−2^ s^−1^, but not at higher irradiances. Lipid accumulation at these growth conditions was thus evaluated by Nile red staining [37]. As reported in Additional file 1: Figure S4B an increased lipid accumulation on a dry weight basis was evident in *e8* mutant at all the different irradiances of growth, with the exception of the highest one, 1500 μmol photons m^−2^ s^−1^. Lipid content per volume of culture was thus increased in *e8* mutant grown at 60, 200 and 400 μmol photons m^−2^ s^−1^ compared to the WT case (Additional file 1: Figure S4C). As reported in Additional file 1: Figure S4 a 295%, 100% and 34% increase in Nile red staining was measured, respectively, at 60, 200 and 400 μmol photons m^−2^ s^−1^compared to WT. The increased lipid accumulation phenotype of *e8* mutant was thus more evident at lower irradiances, suggesting a role of light availability on the lipid accumulation phenotype observed. Interestingly, on a volume basis *e8* mutant grown at 400 μmol photons m^−2^ s^−1^ was accumulating a similar level of lipid compared to the WT case grown at 1500 μmol photons mm^−2^ s^−1^ (Additional file 1: Figure S4). Fatty acid accumulation and productivity were then analyzed at 400 μmol photons m^−2^ s^−1^, this light intensity being sufficient to essentially reach the maximum Nile red staining on a volume basis in *e8* mutant. In particular, lipid fractions of WT and *e8* mutant strain were explored by GC analysis of the total acyl lipid as Fatty acid methyl esters (FAME). As reported in Fig. 4, *e8* mutant was characterized by a ~ 80% increase of FAME accumulation and daily productivity compared to WT on a volume basis. Accordingly, FAME fraction on total biomass was increased by ~ 60% in *e8* mutant compared to the WT case (Fig. 4e), while on a cell basis the FAME accumulation of *e8* mutant was increased by 115%. As reported in Fig. 4g, palmitic acid (C16:0) and palmitoleic acid (C16:1) were the major fatty acids accumulated in both WT and *e8* mutant, with a strong increase being observed in the latter. Moreover, myristic acid (C14:0), stearic acid (C18:0), oleic acid (cis C18:1), elaidic acid (trans C18:1) and linoleic acid (C18:2) were also strongly increased in *e8* mutant compared to WT on a volume basis. Interestingly, the strongest increase was observed in the case of the saturated stearic acid (C18:0) and palmitic acid (C16:0) with a more than twofold increase in *e8* compared to WT.

Lipids production in *N. gaditana* is triggered upon nitrogen deficiency, where metabolism is switched accumulating nitrogen-free lipids [5, 10, 38]. The influence of nitrogen starvation on the FAME accumulation properties of *e8* mutant compared to WT was thus investigated: reduced nitrogen source (nitrate) was thus removed at the end of the growth curve reported in Fig. 4a, in order to boost lipid biosynthesis [5, 10, 38]. A slight reduction of total biomass yield was evident in nitrogen deplete condition (-N) in *e8* mutant compared to WT (Additional file 1: Figure S5A), while a more evident difference was measured in the case of lipid fraction (Additional file 1: Figure S5B). In -N condition the WT strain induced a strong accumulation of fatty acids as previously reported [19, 39], especially palmitic acid (C16:0), palmitoleic acid (C16:1), stearic acid (C18:0), linoleic acid (C18:2) and oleic acid (C18:1 CIS), while no significant increase of total FAME was evident in *e8* mutant in −N compared to nitrogen-replete (+N) condition (Additional file 1: Figure S6). *e8* mutant is thus more productive in terms of lipid accumulation in nitrogen-replete conditions, but it is not able to further increase its lipid fraction in nitrogen deficiency.

### Light distribution is improved in *e8* mutant

**Fig. 5:**
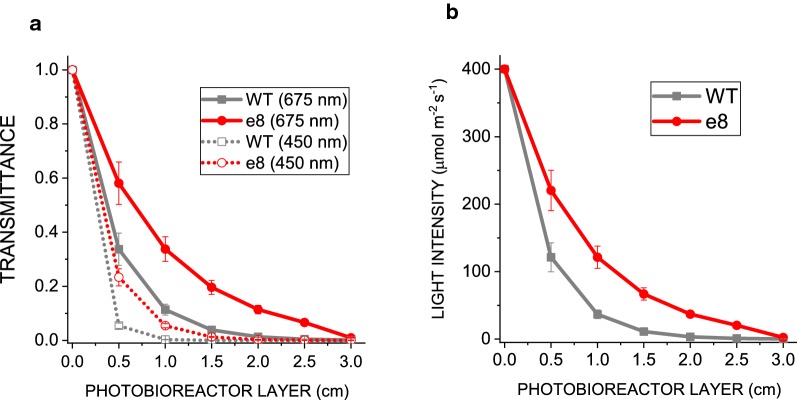
Transmittance and light penetration in photobioreactors. Transmittance (**a**) and irradiances (**b**) at different layer of photobioreactors were calculated considering the absorption spectra of whole cells, the incident light intensity (400 µmol photons m^−2^s^−1^) and the concentration of chlorophyll at exponential phase in photobioreactors. Error bars are reported as standard deviation (*n* = 3)

The results obtained demonstrate that the reduced Chl/cell ratio and the improved light distribution observed yielded an increased lipid productivity in +N conditions. Light distribution in the photobioreactors herein adopted was thus estimated considering the chlorophyll concentration measured in photobioreactors at the exponential phase, the absorption spectra of whole cells in the 400–700 region, the irradiance arriving at the surface of photobioreactor (400 µmol photons m^−2^ s^−1^) and its diameter (3 cm). As reported in Fig. 5a and in Additional file 1: Figure S7, the transmittance at 675 nm and 450 nm, the main peaks of chlorophyll *a* absorption, was higher in the *e8* mutant compared to WT: the transmittance being the ratio between the light not being absorbed or reflected by the sample and the incident light, it was possible to calculate the irradiance arriving at the center of photobioreactor (1.5 cm) considering an incident irradiance of 400 μmol photons m^−2^ s^−1^. As reported in Fig. 5b, *e8* mutant was exposed to a sixfold higher irradiance at the center of the photobioreactor compared to the WT case.

### Genetic characterization of the *e8* mutant strain

*e8* mutant strain was investigated at genetic level to identify those mutations putatively responsible for the phenotypic traits observed. Whole genome Illumina sequencing was performed for WT and *e8*, using the reference genome available for *N. gaditana* for reads alignment and genome assembly [17]. As reported in Additional file 1: Table S1, a 20× coverage was obtained for at least 95% of the genome of both WT and mutant strain. The comparison between WT and *e8* allowed to identify the single nucleotide polymorphisms (SNP) induced in mutant strain by EMS treatment. In particular, *e8* resulted to be mutated in 234 genes, among which 113 genes with not-silent mutations (Table 2, Additional file 2: Dataset S1). The high number of mutations identified complicates the association of the phenotypes observed with the genetic traits of the *e8* mutant. Mutated genes were grouped according to their Gene Ontology (GO) terms and clustered using GO slim terms of plant: as reported in Additional file 1: Figure S8 several biological processes, molecular functions and cellular component were potentially affected by mutations. Among the different mutations, genes encoding for chloroplast located proteins were investigated in order to find possible mutations at the base of the reduced chlorophyll content phenotype: chloroplast transit peptides were predicted using HECTAR software [40] identifying only 4 mutated genes for putative plastid located proteins (Additional file 1: Table S2). In particular, a plastid chaperone protein with a DnaJ domain (Naga_100340g1) was mutated: protein subunits with DnaJ domains have been previously reported to be involved in plastids in several processes ranging from biogenesis of thylakoid membranes, translation, to mRNA stability [41]. Other mutations on chloroplast targeted proteins were on genes encoding for NHL-repeat protein (Naga_100040g45), for a dehydrogenase reductase SDR (short-chain dehydrogenase/reductase) -family protein (Naga_100641g4) and a protein of unknown function (Naga_100008g127). Homologous proteins in the case for NHL-repeat protein (Naga_100040g45) and dehydrogenase reductase SDR-family protein were reported to be, respectively, involved in biotic and abiotic stresses [42] and in the secondary metabolism [43] but their possible correlation with the phenotype observed in *e8* mutant is not obvious. Among the SNPs identified in gene upstream regions, possibly affecting gene expression, a mutation was identified in a Photosystem II s4 domain protein (Naga_100303g8). Photosystem II s4 domain protein in the cyanobacterium *Synechocystis* sp. *PCC 6803* has been reported to be involved in balancing photosynthetic electron transport [44]. However, the similar PSII quantum yield (F_v_/F_m_) observed in WT and *e8* mutant suggests that impact of the mutation in this Photosystem II s4 domain protein is minor.

**Table 2:** Statistics of the mutations found in *e8* mutant strain

Total SNPs	Predicted effect			
	Low	Moderate	High	Other
240	36	80	1	123SNPs

Total number of SNPs found is reported in the first column. The predicted effect of mutations are reported according to SNPeff software: HIGH is for mutation probably causing protein truncation, loss of function or triggering nonsense-mediated decay, Moderate are non-disruptive variants that might change protein effectiveness and LOW are mutations harmless or unlikely to change protein behavior. SNPs with predicted non-coding variants or variants affecting non-coding genes, where predictions are difficult or there is no evidence of impact were not considered: the number of remaining SNPs is reported in the last column (Other)

Other mutated genes found in *e8* mutant possibly linked with the reduced chlorophyll content per cell observed include genes involved in regulation of gene expression, as a CCT (CONSTANS, CO-like and TOC1) domain containing protein (Naga_100027g10). This gene in higher plants is involved in control of flowering and heading [45, 46] and could thus be putatively involved in the regulation of chlorophyll biosynthesis and/or plastid morphology. Other mutations identified on a transcription elongation factor (Naga_100012g30), a regulator of chromosome condensation (Naga_100664g1) and a ribosomal RNA small subunit methyltransferase B (Naga_100044g6) could generally lead to altered gene expression and protein synthesis. Since the selected mutant showed an increased lipid content per cell, mutations affecting lipid metabolism were also investigated: mutations in a beta-ketoacyl synthase (Naga_100086g24), 24-dehydrocholesterol reductase (Naga_100012g52) and digalactosyldiacylglycerol synthase 1 (*dgd1*, Naga_100010g107) were identified. The beta-ketoacyl synthase (Naga_100086g24) and 2,4-dehydrocholesterol reductase are involved in lipid biosynthesis [39, 47], and the correlation between mutations in these genes and the observed phenotypes in *e8* is not obvious. More interesting is the case of the gene encoding for the digalactosyldiacylglycerol synthase 1 (*dgd1*, Naga_100010g107): this gene is mutated in the CDS region, leading to the substitution of a proline residue with a serine. Thus, the mutation introduced caused the substitution of an aliphatic residue with a polar one, potentially affecting the enzymatic activity of the protein. A *dgd1* mutant of *Arabidopsis* was previously isolated showing a reduction of 90% of DGDG content and strong reduction in chlorophyll content per leaf area [48]. Consistently with a reduced activity of DGD1 enzyme, a strong reduction of C20:5 fatty acid (EPA) on total lipid fraction was evident in *e8* mutant either in +N or -N conditions (Additional file 1: Information Figure S6B). C20:5 has been indeed previously reported in *N. gaditana* to be the major constituents of MGDG and DGDG being found almost essentially in these lipids in +N conditions [19]. For these reasons, in the specific case of *N. gaditana*, the quantification of C20:5 fatty acid can be used as a proxy of MGDG and DGDG accumulation, demonstrating a reduced content of the main thylakoidal lipids in *e8* mutant compared to the WT case.

## Discussion

In this work, mutants with reduced chlorophyll content per cell and increased lipid productivity were screened upon random chemical mutagenesis in *N. gaditana*. The strategy to improve productivity reducing the chlorophyll content per cell has been reported for several microalgae as *N. gaditana* [22], *Chlamydomonas reinhardtii* [24, 49, 50], *Chlorella sorokiniana* [23]*, Chlorella vulgaris* [51] and cyanobacteria [32] among others. Generation of mutants by chemical mutagenesis presents the advantage to produce strains not considered as GMO (genetically modified organism), and more easily cultivatable in outdoor systems without the restrictive authorizations required for GMO strains in several countries [52]. Functional PSII antenna size and light-dependent oxygen evolution were not altered in pale green mutant *e8*, indicating a more general reorganization of plastid assembly in this mutant leading to a similar functioning of the photosynthetic apparatus on a chlorophyll basis, despite the reduction in total chlorophyll per cell. In line with this finding *e8* mutant did not present any mutations on light harvesting subunits. Unaltered photosynthetic efficiency in pale green mutant has been previously reported in several microalgae species [22, 23, 31, 47–49], chlorophyll content per cell being not necessarily influencing the functionality of the photosynthetic apparatus, but rather being possibly linked to chloroplast biogenesis. Consistently *e8* mutant was characterized by a strong reduction in C20:5 fatty acid accumulation, this lipid being the main constituent of thylakoid glycerolipids MGDG, DGDG and SQDG in *N. gaditana* [19].

*e8* mutant was characterized by an increased biomass productivity compared to the WT case at low-medium light, while at saturating irradiances an increased lipid accumulation was rather observed (Fig. 4, Additional file 1: Figure S4). Since lipids are a class of macromolecules with the highest energy density, an increased lipid accumulation implies an improved light energy conversion efficiency. Considering the irradiance-dependent phenotype and the similar photosynthetic properties compared to the WT case (Figs. 3, 5, Additional file 1: Figure S4), the improved photosynthetic efficiency at the base of the increased lipid content in *e8* mutant is thus related to the improved light penetration in the photobioreactor and more homogenous light availability due to the reduced chlorophyll content per cell observed in the mutant (Table 1; Fig. 5). Accordingly, the zeaxanthin content measured in *e8* mutant was increased compared to WT (Table 1): zeaxanthin accumulation is indeed triggered in high light, providing other evidences for the increased penetration of light in photobioreactors in the case of *e8* mutant cultivation. In *N. gaditana* lipid production is triggered by high light [39]: the improved light distribution in *e8* mutant could thus be the major reason for the increased lipid productivity observed in the *e8* mutant. In order to elucidate the genetic base of the reduced chlorophyll content per cell phenotype and to investigate other possible genetic traits associated to the increased lipid content phenotype whole-genome sequencing was performed and mutations on 234 genes were identified, among which 113 in coding regions. Interestingly, *e8* mutant present a non-conservative mutation of *dgd1* gene, encoding for a key enzyme involved in DGDG biosynthesis. DGDG and MGDG are the major lipids of photosynthetic membranes. *dgd1* mutant of *Arabidopsis* showed an impaired DGDG synthesis with a rearrangements in all pigment-protein complexes [48]. In plants and in algae DGDG is synthesized in at least two parallel pathways, the ‘prokaryotic pathway’, restricted to plastid, and the ‘eukaryotic pathway’ which involves both plastid and endoplasmic reticulum [48, 53]. The *dgd1* gene mutated in the *e8* mutant encodes for an enzyme involved in the ‘eukaryotic pathway’. Mutation in *dgd1* gene in *A. thaliana* caused a reduction in DGDG biosynthesis for thylakoid assembly and pale green leaves [54]. Thus, we suggest that mutation in *dgd1* gene in *e8* mutant is responsible for the reduction in chlorophyll content per cell. The increased lipid accumulation in *e8* mutant could be a consequence of an improved light availability experienced by the mutant strain compared to WT (Fig. 5), because of the reduced cells pigmentation. This consideration is consistent with previous observations about increased lipid accumulation in *N. gaditana* upon exposure to high irradiances [38, 39, 55]. Similar effects of high irradiances on lipid biosynthesis were reported also in the case of *Nannochloropsis oceanica* and *Phaeodactylum tricornutum* [56]. Alternatively, the high lipid accumulation observed in the case of *e8* could be a consequence of a re-direction of fatty acid metabolism due to altered glycerolipid accumulation: this is however unlikely, considering similar lipid accumulation in the *dgd1* mutant of *A. thaliana* [48].

However, the mutations introduced in *e8* mutant have a side effect in nitrogen starvation: in -N condition, the increased lipid production trait was lost in *e8*. In *N. gaditana* nitrogen starvation induces a lipid accumulation through the degradation of existing membrane lipids (MGDG and DGDG mainly) and in part by the de novo synthesis of TAG [19]. In *e8* mutant, due to the mutation in *dgd1* gene, membrane glycerolipids are likely kept to a minimum level sufficient to avoid impairment of photosynthetic membrane integrity, allowing for a sustainable photoautotrophic growth, but the reduced pool of thylakoid membrane lipids impairs the lipid boost observed in nitrogen starvation.

Whole-genomic sequencing of the mutant revealed that it was characterized by several SNPs: this is a disadvantage in using chemical mutagenesis to produce strains with phenotypic traits of interest, making the correlation between genotype and phenotype extremely difficult and increase the possibility of unexpected phenotypes in some peculiar conditions. RNA-seq analysis could also provide additional information in order to interpret the phenotypic traits observed in *e8* mutant.

Mutant complementation or specific mutagenesis with homologous recombination or genome editing will allow to prove the correlation between specific mutations and the observed phenotypes. The mutations introduced leading to reduced pigmentation and improved lipid productivity could then be considered to possibly extend these phenotypic traits in other microalgal species.

## Conclusions

The characterization of the biomass and lipid production of *N. gaditana e8* mutant demonstrate that reduced chlorophyll content per cell could be a convenient trait to be selected for improving lipid production in nitrogen-replete conditions. Indeed, the selected mutant exhibited an increased lipid productivity in +N condition of ~ 80% compared to WT on a volumetric base. This trait is interesting considering the strong increased in C16:0, C16:1, C18:0 and C18:1 without the energetic and economic costs of inducing nutrient starvation, and their possible use for biodiesel production [57, 58]. Improved photosynthetic efficiency by manipulating chlorophyll per cell content is thus a suitable strategy to increase lipid productivity in *N. gaditana.* Reduced chlorophyll per cell phenotype can be obtained by chemical mutagenesis, as reported in this work, or by specific genetic manipulation by homologous recombination [59] or genome editing [30, 60]. Direct genetic engineering would have also allow reducing the risk of introducing additional mutations with possible negative side effects.

## Methods

### Culture conditions, mutagenesis and mutant selection

*N. gaditana* WT (CCAP849/5) and mutants were cultivated in sterile filtered f/2 medium [61] modified as described in Alboresi et al. 2016. Cells were grown at temperature of 24 ± 1 °C, in a 16 h light/8 h dark photoperiod with a fluorescent light of about 70 µmol photons m^−2^ s^−1^ (low light = LL). Cells numbers were monitored with a Bürker Counting Chamber (HBG, Germany) under light microscope. Chemical mutagenesis was induced using the mutagenic agent ethyl methane sulfonate (EMS) as described in the following: EMS was added to 10 [8] cell/ml at concentrations of 0.75%, 1.5%, 2% and 2.5%. Samples were incubated for 2 h in dark and then diluted in 10% sodium thiosulfate solution inactivating the EMS activity. Cells were then centrifuged, washed twice with 1 M NaCl, dissolved in 500 µl of f/2 medium and kept overnight under low light. Cells were then plated on f/2 solid medium and kept under control light for at least 2 weeks. The cells treated with EMS concentration inducing a 95% of mortality was used for the following screening procedure. Pale green mutants were selected on the base of visible phenotype. Selected colonies were cultured in liquid f/2 medium and the chlorophyll content per cell was estimated by measuring absorption of whole cells at 680 nm and at 730 nm: strains with at least a 25% reduced 680/730 absorption ratio were selected. Further screening was performed measuring the chlorophyll content per cell and Nile red staining as described below and in the Results section.

### Nile red staining

Lipid content by Nile red staining was evaluated as previously reported [37].

### Measurement of photosynthetic parameters

In vivo chlorophyll fluorescence was measured with Dual PAM-100 fluorometer (Walz, Effeltrich, Germany) at room temperature (RT) using a saturating light at 6000 µmol photons m^−2^ s^−1^ and actinic light of 1500 µmol photons m^−2^ s^−1^. The NPQ parameter was calculated form the maximum fluorescence induced by a saturating pulse in the dark (F_M_) or after actinic light exposure (F_M_’) as (F_M_–F_M_′)/F_M_′. Proton motive force upon exposure to different light intensities was measured by Electrochromic shift (ECS) with MultispeQ v2.0 (PhotosynQ) according to [62].

PSII functional antenna size was measured following kinetic of PSII fluorescence emission in cells treated with 1 × 10^− 5^ M 3-(3,4-dichlorophenyl)-1,1-dimethylurea (DCMU). PSII antenna size is inversely proportional to the time required for reaching 2/3 of the maximum fluorescence emission [33].

Oxygen evolution curves were performed as described [63]. Net oxygen production was calculated subtracting the oxygen consumption in the dark after each measurement at the different actinic lights. Experimental data were fitted with hyperbolic functions in order to retrieve the Pmax (maximum photosynthetic activity) and half saturation light intensity values (light intensity at which the oxygen evolved is half of Pmax).

### Pigment extraction and analysis

The chlorophyll *a* and total carotenoids were extracted from *N. gaditana* with 100% DMSO at 60 °C for 24 h in dark conditions and analyzed by HPLC as described in [64]. De-epoxidation index was calculated as (zea + anthera/2)/(anthera + viola + zea).

### Biomass and lipid productivity

Biomass productivity of WT and *e8* mutant was evaluated in small photobioreactors (80 ml) in Multi-Cultivator MC1000 system (Photon System Instrument, Czech Republic) at 24 °C under continuous light at 60, 200, 400 or 1500 µmol m^−2^ s^−1^ as described in the Results section. Biomass accumulation was evaluated considering the dry weight per volume (g/L) obtained at the end of the growth curve. Maximum daily productivity (gr L^−1^ day^−1^) was determined at the exponential phase of growth curve. Fatty acid methyl esters (FAME) were measured at the end of the exponential phase as reported in [51]. Lipid and fatty acids accumulation were expressed on a volume base (mg/L) or as a fraction of biomass dry weight (mg/gr). Daily lipid productivity was calculated from lipid content and the time (days) at which the lipid analysis was performed.

### Sequencing and computational analysis

Sequencing of mutant and WT strain was carried out on an Illumina NextSeq and an Illumina HiSeq 1000, respectively. The raw reads resulting from the sequencing were processed using Scythe [65] and Sickle [66] to remove Illumina adapters and low-quality reads. All sequences were mapped to the *N. gaditana* B-31 assembly [17] using the Burrows–Wheeler Aligner (BWA) [67]. Deduplication and indel realignment were performed with PicardTools [68]. Variants were identified using three softwares: GATK [69], Freebayes v1.3 [70] and breseq v0.35.1 [71]. Variants were quality filtered (DP > 5 and QUAL > 30) and for each sample only mutations identified by three out of three variant callers were selected. Variants found in both samples were then discarded. Prediction of SNPs effect was performed using SNPeff software [72]. The dataset of SNPs identified is reported in Additional file 2: Dataset S1. Only SNPs not predicted with MODIFIER effect, thus only SNPs located elsewhere than upstream or downstream of a gene, 5′ or 3′ UTR regions or intergenic regions were considered for the following analysis. Targeting prediction was performed using HECTAR [40]. GO analyses were performed on Blast2go [73], using Blast2go GO term grouped using plant slim subset and eventually visualized with REVIGO [74] in base of the number of gene for each GO term.

## Supplementary information

**Additional file 1: Table S1.** Coverage obtained by Illumina sequencing for WT and *e8* mutant strain. **Table S2.** Mutation identified on gene coding for proteins putatively located in the chloroplast. Prediction of chloroplast transit peptide was performed with Hectar software. **Figure S1.** Mutants screening by 680/730 nm absorption ratio. Absorption ratio 680/730 nm was used to assay the chlorophylls per cell content. Only colonies with a reduction of at least 25% was selected for further analyses. Error bars are reported as standard deviation (n=3). **Figure S2.** Light dependent zeaxanthin accumulation in WT and *e8*. Samples were illuminated for 1 h with a strong light (2500 μmol photons m-2 s-1). Pigments composition was evaluated at different time points by DMSO extraction and HPLC analysis. (A) Depoxidation index calculated as (zea + anthera/2)/(anthera+viola+zea). (B) Zeaxanthin per carotenoid content. (C) Zeaxanthin per chlorophyll content. Errors are reported as standard deviation, significantly different values are marked with * if p<0.05 and ** if p<0.01, as determined by unpaired two-sample t-test (n=3). **Figure S3.** Chlorophyll bleaching in wild type and *e8* mutant strain exposed to strong light. Chlorophyll bleaching kinetics of WT and *e8* mutant strains were determined measuring the decrease of chlorophyll absorption upon exposure to 2500 μmol photons m-2 s-1. Errors are reported as standard deviation, (n=3). The statistical analysis of the results obtained was performed by unpaired two sample t-test revealing no statistically significant difference being p-values > 0.1 at the different time points. **Figure S4.** Biomass and lipid productivity of WT and e8 mutant at different irradiances. (A)) Maximum daily productivity in terms of gr L-1 day-1. (B, C) Nile red fluorescence of WT and e8 mutant normalized to dry weight (B) or to the culture volume (C). (D) Fold change of Nile red fluorescence and biomass dry weight on a volumetric base in e8 mutant compared to WT. Errors are reported as standard deviation, significantly different values are marked with * if p<0.05 and ** if p<0.01, as determined by unpaired two sample t-test (n=3). 60. **Figure S5.** Dry weight and FAME content in WT and e8 mutant in nitrogen starvation. Dry weight(A) and FAME content (B) in cells grown in nitrogen deplete medium for WT and *e8* mutant strain. Errors are reported as standard deviation, the statistical significance of differences between WT and e8 is indicated as ** (p<0.01), as determined by unpaired two-sample t-test (n=3). **Figure S6.** Acyl chain composition of lipid fraction from WT and *e8* mutant in nitrogen replete conditions (+N) or after nitrogen starvation (-N). (A) fatty acid content per liter of culture. (B) Fold change of fatty acid fraction on total fatty acids content in e8 normalized to the WT case. Errors are reported as standard deviation, statistically significantly different values between WT and e8 in (A) and values statistically significantly different than 1 in (B) are marked with * if p<0.05 and ** if p<0.01, as determined by unpaired two sample t-test (n=3). **Figure S7.** Visible light transmittance in photobioreactors at different layers for WT and *e8* mutant cultures. **Figure S8.** GO slim terms of mutated genes of *e8*. The GO terms were restricted to GO slim terms of plant for an easier visualization. Each dot is proportional with the number of genes related to a specific category of GO terms (max 30 genes, min 1 genes).

**Additional file 2: Dataset S1.** List of single nucleotide variants (SNVs) identified in *e8* mutant.

## Data Availability

The datasets supporting the conclusions of this article are included within the article and its Additional files 1, 2. Sequenced data discussed in this work have been submitted to the Sequence Read Archive (SRA) repository of the NCBI database and are available under Bioproject accession number PRJNA623339.

## Abbreviations

Chl: Chlorophyll
DCMU: 3-(3,4-Dichlorophenyl)-1,1-dimethylurea
DGDG: Digalactosyldiacylglycerol
*dgd1*: Digalactosyldiacylglycerol synthase 1
EPA: Eicosapentaenoic acid
FAMEs: Fatty acid methyl-esters
NPQ: Non-photochemical quenching
PSII: Photosystem II
TAGs: Triacylglycerols
