# Supplementary material for: Improved lipid productivity in Nannochloropsis gaditana in nitrogen-replete conditions by selection of pale green mutants
Source: Biotechnol Biofuels. 2020 Apr 21;13:78. doi: 10.1186/s13068-020-01718-8 (PMC7175523; doi:10.1186/s13068-020-01718-8)

ADDITIONAL FILE 1

Table S1. Coverage obtained by Illumina sequencing for WT and *e8* mutant strain.

	WT	***e8***
mean coverage	115.02	72.42
% 1X cov	98.79	98.81
% 5X cov	98.47	98.63
% 10X cov	98.01	98.10
% 20X cov	96.92	95.77

Table S2. Mutation identified on gene coding for proteins putatively located in the chloroplast. Prediction of chloroplast transit peptide was performed with Hectar software.

**Mutant**	**Gene**	**Mutation**	**Annotation**
e8	Naga_100008g127	3_prime_UTR_variant	Protein of unknown function DUF1118
e8	Naga_100040g45	missense_variant	nhl repeat containing protein 2
e8	Naga_100340g1	missense_variant	chaperone protein
e8	Naga_100641g4	missense_variant	dehydrogenase reductase sdr family member 9

Supplementary Figure S1. Mutants screening by 680/730 nm absorption ratio. Absorption ratio 680/730 nm was used to assay the chlorophylls per cell content. Only colonies with a reduction of at least 25% was selected for further analyses. Error bars are reported as standard deviation (n=3).

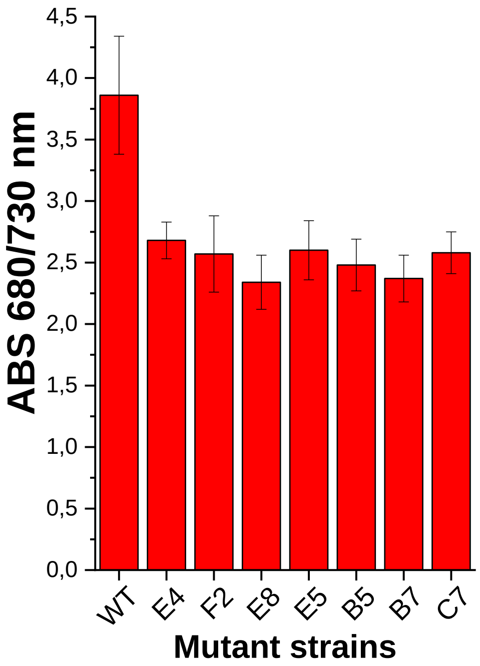

Supplementary Figure S2. Light dependent zeaxanthin accumulation in WT and *e8*. Samples were illuminated for 1 h with a strong light (2500 µmol photons m^{-2} s^{-1}). Pigments composition was evaluated at different time points by DMSO extraction and HPLC analysis. (A) Depoxidation index calculated as (zea + anthera/2) /(antera+viola+zea). (B) Zeaxanthin per carotenoid content. (C) Zeaxanthin per chlorophyll content. Errors are reported as standard deviation, significantly different values are marked with * if p<0.05 and ** if p<0.01, as determined by unpaired two-sample t-test (n=3).

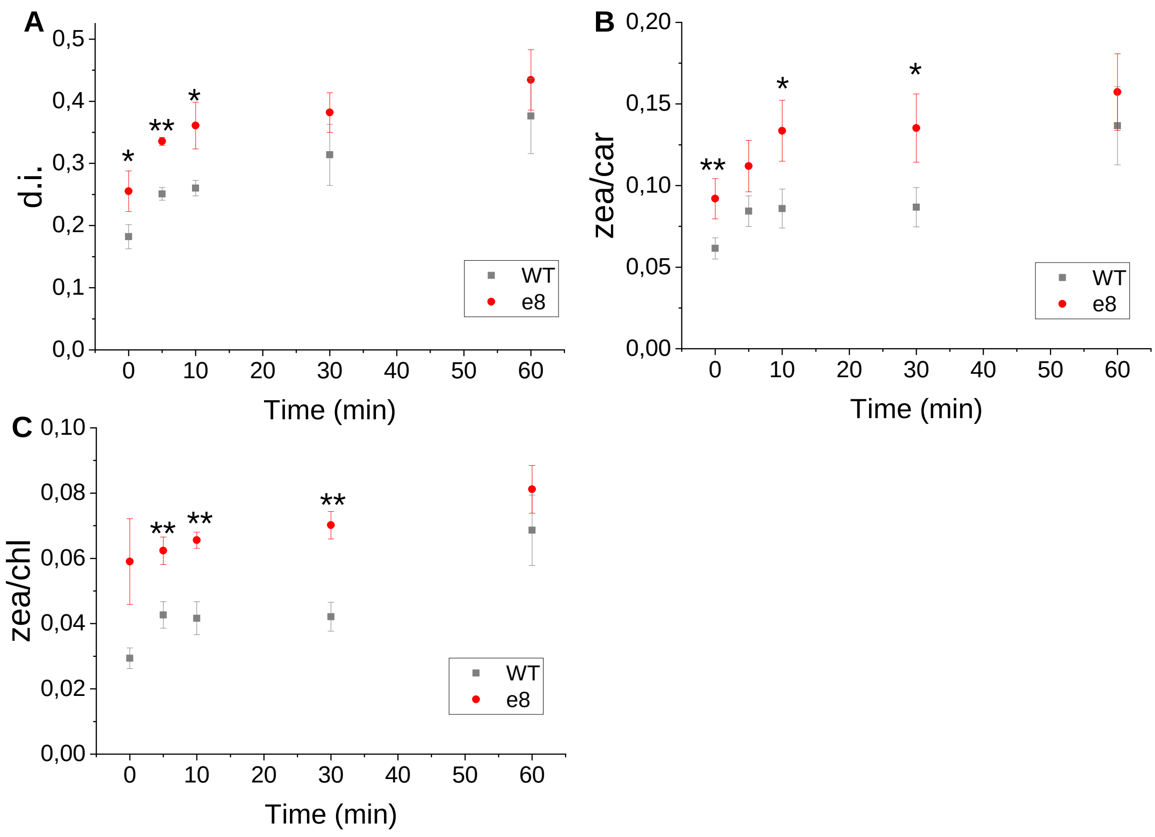

Supplementary Figure S3. Chlorophyll bleaching in wild type and *e8* mutant strain exposed to strong light. Chlorophyll bleaching kinetics of WT and *e8* mutant strains were determined measuring the decrease of chlorophyll absorption upon exposure to 2500 μmol photons m^{-2} s^{-1}. Errors are reported as standard deviation, (n=3). The statistical analysis of the results obtained was performed by unpaired two sample t-test revealing no statistically significant difference being p-values > 0.1 at the different time points.

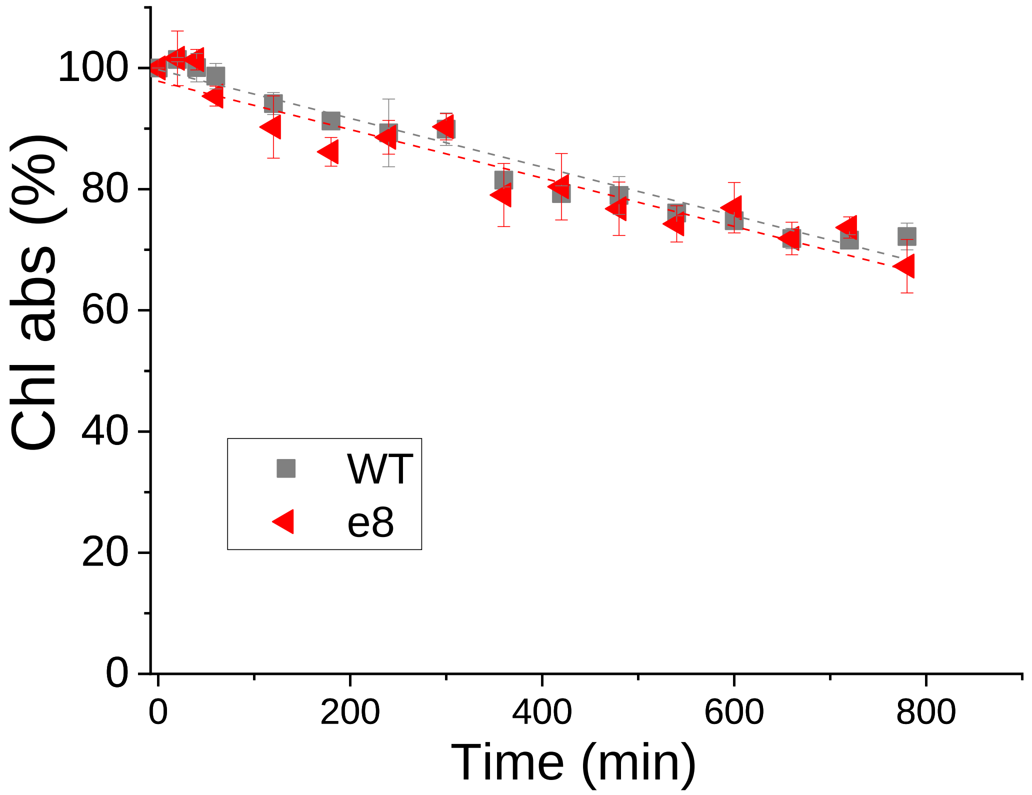

Supplementary Figure S4. Biomass and lipid productivity of WT and e8 mutant at different irradiances. (A)) Maximum daily productivity in terms of gr L^{-1} day^{-1}. (B, C) Nile Red fluorescence of WT and e8 mutant normalized to dry weight (B) or to the colture volume (C). (D) Fold change of Nile Red fluorescence and biomass dry weight on a volumetric base in e8 mutant compared to WT. Errors are reported as standard deviation, significantly different values are marked with * if $p<0.05$ and ** if $p<0.01$, as determined by unpaired two sample t-test (n=3).

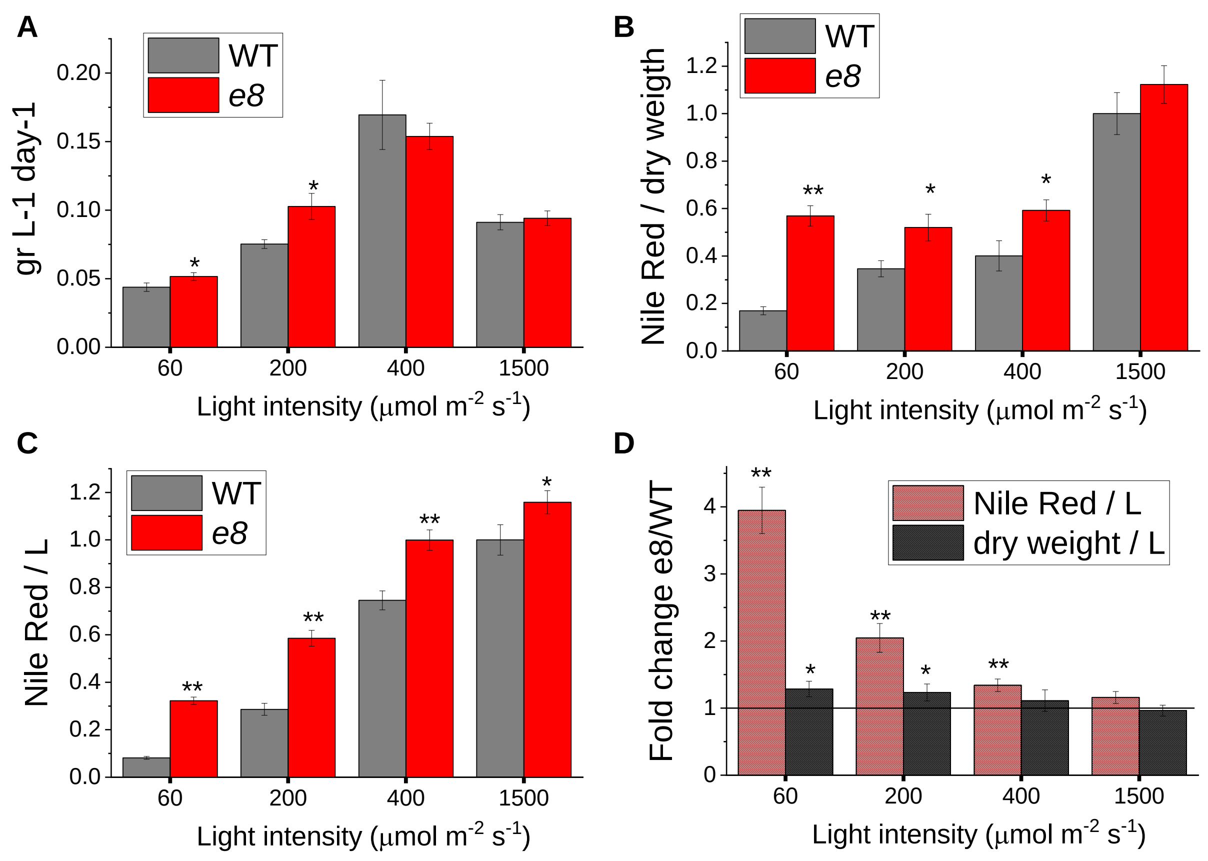

Supplementary Figure S5. Dry weight and FAME content in WT and e8 mutant in nitrogen starvation. Dry weight(A) and FAME content (B) in cells grown in nitrogen deplete medium for WT and *e8* mutant strain. Errors are reported as standard deviation, the statistical significance of differences between WT and e8 is indicated as ** ($p<0.01$), as determined by unpaired two-sample t-test (n=3).

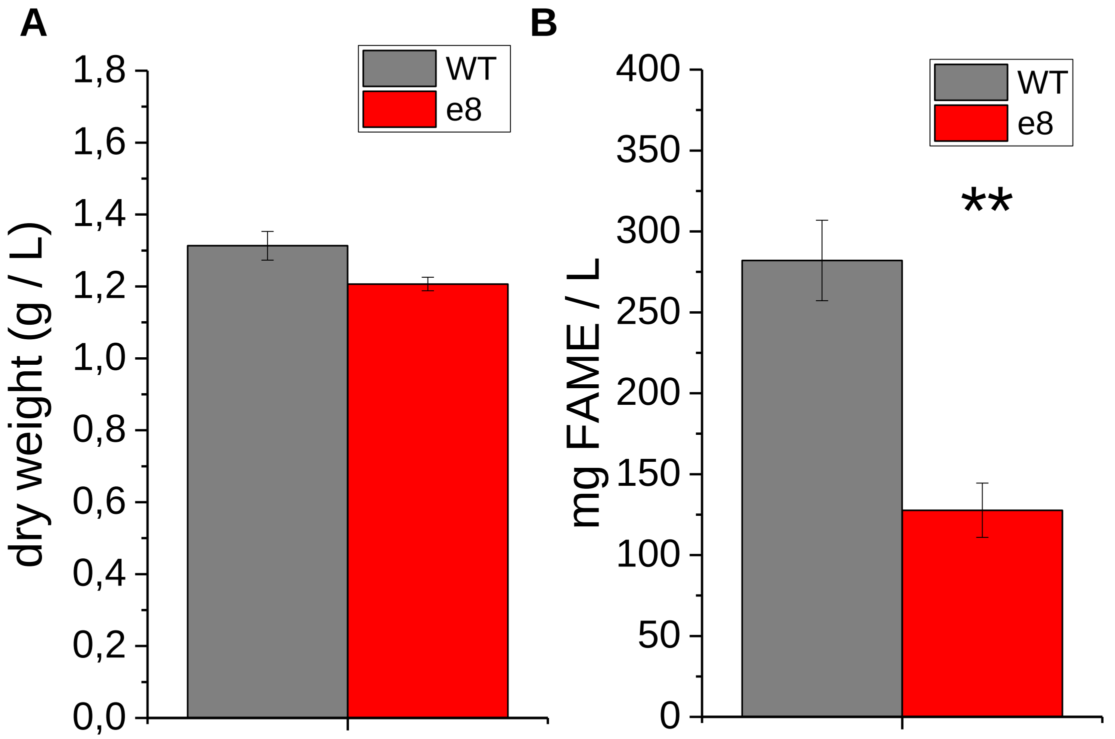

Supplementary Figure S6. Acyl chain composition of lipid fraction from WT and *e8* mutant in nitrogen replete conditions (+N) or after nitrogen starvation (-N). (A) fatty acid content per liter of culture. (B) Fold change of fatty acid fraction on total fatty acids content in e8 normalized to the WT case. Errors are reported as standard deviation, statistically significantly different values between WT and e8 in (A) and values statistically significantly different than 1 in (B) are marked with * if $p<0.05$ and ** if $p<0.01$, as determined by unpaired two sample t-test (n=3).

A

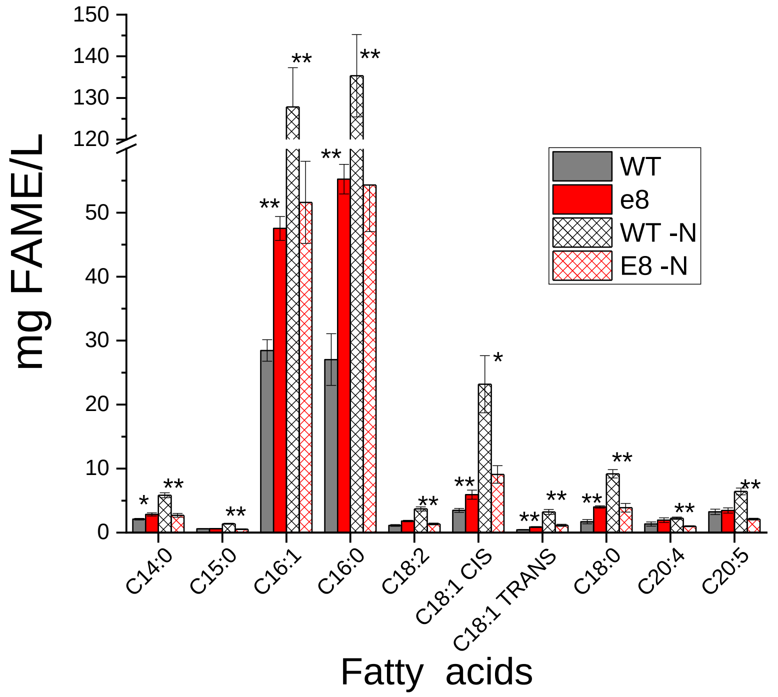

B

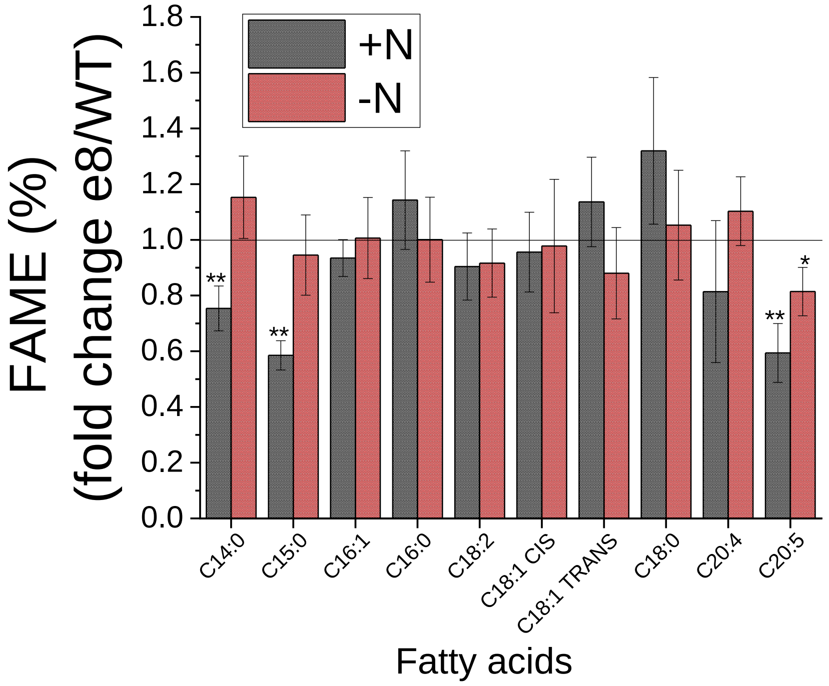

Supplementary Figure S7. Visible light transmittance in photobioreactors at different layers for WT and *e8* mutant cultures.

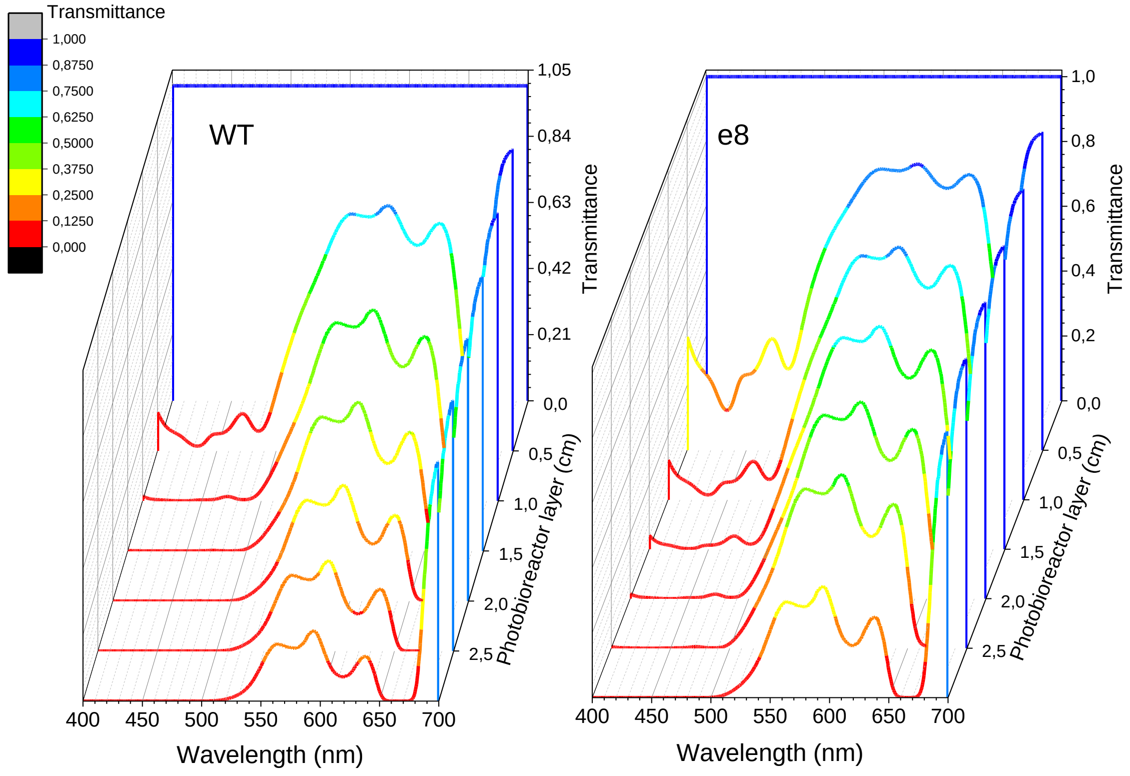

Supplementary Figure S8. GO slim terms of mutated genes of *e8*. The GO terms were restricted to GO slim terms of plant for an easier visualization. Each dot is proportional with the number of genes related to a specific category of GO terms (max 30 genes, min 1 genes).

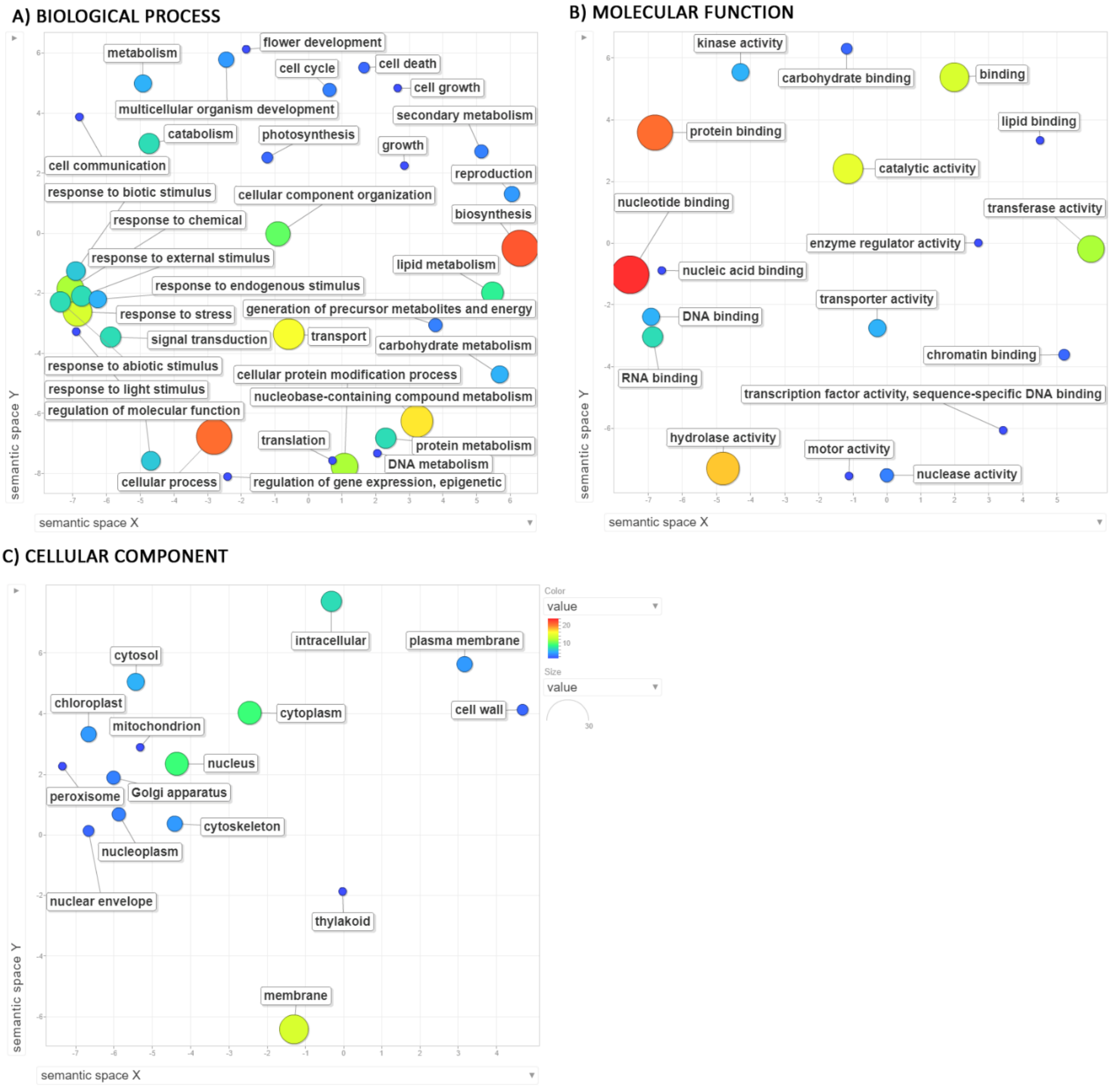

Supplement: Supplementary file 1 — Additional file 1: Table S1. Coverage obtained by Illumina sequencing for WT and e8 mutant strain. Table S2. Mutation identified on gene coding for proteins putatively located in the chloroplast. Prediction of chloroplast transit peptide was performed with Hectar software. Figure S1. Mutants screening by 680/730 nm absorption ratio. Absorption ratio 680/730 nm was used to assay the chlorophylls per cell content. Only colonies with a reduction of at least 25% was selected for further analyses. Error bars are reported as standard deviation (n=3). Figure S2. Light dependent zeaxanthin accumulation in WT and e8. Samples were illuminated for 1 h with a strong light (2500 μmol photons m-2 s-1). Pigments composition was evaluated at different time points by DMSO extraction and HPLC analysis. (A) Depoxidation index calculated as (zea + anthera/2)/(anthera+viola+zea). (B) Zeaxanthin per carotenoid content. (C) Zeaxanthin per chlorophyll content. Errors are reported as standard deviation, significantly different values are marked with * if p<0.05 and ** if p<0.01, as determined by unpaired two-sample t-test (n=3). Figure S3. Chlorophyll bleaching in wild type and e8 mutant strain exposed to strong light. Chlorophyll bleaching kinetics of WT and e8 mutant strains were determined measuring the decrease of chlorophyll absorption upon exposure to 2500 μmol photons m-2 s-1. Errors are reported as standard deviation, (n=3). The statistical analysis of the results obtained was performed by unpaired two sample t-test revealing no statistically significant difference being p-values > 0.1 at the different time points. Figure S4. Biomass and lipid productivity of WT and e8 mutant at different irradiances. (A)) Maximum daily productivity in terms of gr L-1 day-1. (B, C) Nile red fluorescence of WT and e8 mutant normalized to dry weight (B) or to the culture volume (C). (D) Fold change of Nile red fluorescence and biomass dry weight on a volumetric base in e8 mutant compared to WT. Errors [file 13068_2020_1718_MOESM1_ESM.pdf]
